# The impact of non- and anthracycline-based chemotherapy on fatigue in breast cancer survivors: results from WF-97415

**DOI:** 10.1007/s00520-024-08717-7

**Published:** 2024-07-19

**Authors:** Nancy E. Avis, Beverly J. Levine, Heidi D. Klepin, Shannon L. Mihalko, Peter H. Brubaker, Tonya Moore, Amy C. Ladd, Susan F. Dent, Mary Helen Hackney, Bonnie Ky, William O. Ntim, Lynne I. Wagner, Kathryn E. Weaver, W. Gregory Hundley, the UPBEAT study team

**Affiliations:** 1 Department of Social Sciences and Health Policy, Wake Forest University School of Medicine, Medical Center Blvd., Winston-Salem, NC 27157 USA; 2 Department of Hematology and Oncology, Wake Forest University School of Medicine, Winston-Salem, NC USA; 3 Department of Health and Exercise Science, Wake Forest University, Winston-Salem, NC USA; 4 Section On Cardiovascular Medicine, Department of Internal Medicine, Wake Forest School of Medicine, Winston-Salem, NC USA; 5 Department of Internal Medicine, Division of Cardiology, Pauley Heart Center, Virginia Commonwealth University School of Medicine, Richmond, VA USA; 6 Division of Medical Oncology, Duke Cancer Institute, Duke University, Durham, NC USA; 7 Department of Hematology, Oncology, and Palliative Care, Massey Cancer Center, Virginia Commonwealth University, Richmond, VA USA; 8 Division of Cardiovascular Medicine, University of Pennsylvania, Philadelphia, PA USA; 9 UNC School of Medicine, Novant Health Campus, Novant Heart & Vascular Institute, Charlotte, NC USA; 10 UNC Gillings School of Global Public Health, UNC Lineberger Comprehensive Cancer Center, Chapel Hill, NC USA

**Keywords:** Breast neoplasm, Chemotherapy, Fatigue, Longitudinal, Anthracycline

## Abstract

**Purpose:**

To examine the differential effect of non- and anthracycline-based chemotherapy on fatigue over 12 months post-diagnosis among breast cancer survivors.

**Methods:**

This study is based on a prospective Wake Forest NCI Community Oncology Research Program (NCORP) multicenter cohort study (WF-97415) of women with stage I to III breast cancer and non-cancer controls. Analyses compared those: 1) receiving, or 2) not receiving anthracycline chemotherapy, 3) receiving aromatase inhibitors (AIs) without chemotherapy, with 4) a comparator group without a history of cancer. In-person clinic assessments were conducted at: baseline (prior to chemotherapy or start of AI therapy), and 3 and 12 months after baseline. The Functional Assessment of Chronic Illness Therapy-Fatigue scale was the primary outcome. Estimated least squares means by group using mixed models with a random subject effect, fixed effects of time and group, and the interaction between time and group was used to compare groups across time, controlling for age, comorbidities, and treatment variables.

**Results:**

Among 284 women (mean age = 53.4 years, sd 11.9 years), there was a significant (p < 0.0001) group by time interaction, with a sharp increase in fatigue at 3 months in the two chemotherapy groups in comparison to the non-chemotherapy and non-cancer controls. The two chemotherapy groups did not significantly differ in fatigue at any time point.

**Conclusion:**

Women with breast cancer who receive non- or anthracycline-based chemotherapy experience similar trends in and levels of fatigue within the first year of treatment and greater fatigue than women receiving AIs alone or women without breast cancer.

## Introduction

Fatigue is a frequent and distressing side effect of cancer treatment that negatively affects quality of life [1–5] and can be chronic for many breast cancer survivors (BCS). Prevalence estimates range from 25 to 99% depending on the population, type of treatment, and method of assessment [6]. Numerous studies have evaluated factors related to fatigue during and following treatment and frequently found that treatment-related factors (e.g., chemotherapy, radiation), younger age, more comorbidities, higher body mass index, depression, and lower physical activity are associated with greater fatigue [7–13]. Although chemotherapy is one of the most consistent factors related to fatigue [1, 14, 15], limited literature exists on the impact of different types of chemotherapy on fatigue.

Anthracycline remains an important component of systemic therapy for the treatment of breast cancer, but can be associated with a number of side effects, including fatigue [16]. It is often thought that those who receive anthracycline-based chemotherapy experience greater fatigue than those who receive less aggressive chemotherapy [17, 18], but there are few studies that have examined specific types of chemotherapy in relation to fatigue and they have mixed results [10, 17, 19, 20].

One early study found no difference in reports of fatigue over 2 cycles among women receiving the following chemotherapy regimens for breast cancer: cyclophosphamide and fluorouracil combined with methotrexate (CMF), doxorubicin (CAF), or mitoxantrone (CNF) [19]. Another study, however, comparing three chemotherapy regimens (CMF, CAF, and doxorubicin with cyclophosphamide) found that the chemotherapy protocols containing doxorubicin were associated with greater fatigue, but only after the first cycle [17]. A study that examined subgroups of patients with distinct trajectories of fatigue while undergoing the first three cycles of chemotherapy (AC-T, TC, or TCH) found three trajectories of fatigue: patients with low initial fatigue that increased following infusion, but quickly abated; a transient group that had a pronounced increase in fatigue following infusion and abated more slowly; and a group that consistently showed elevated fatigue [10]. Type of chemotherapy regimen was not related to fatigue trajectory. A more recent study of neoadjuvant chemotherapy among 54 women with early stage breast cancer, compared density of anthracycline cycle, i.e., whether it was given every 2 (dose-dense) or every 3 weeks (standard). Results showed that after 25 weeks of treatment, women who received the dose-dense regimen reported greater physical, but not mental, fatigue [20].

These studies were all small (all fewer than 86 women), and did not control for selection factors related to chemotherapy regimen (e.g., age and comorbidities) or the subsequent administration of radiation therapy. Research has shown that younger female cancer survivors typically report greater fatigue post-treatment [11, 21, 22], and they also typically receive more aggressive treatment [23], thus highlighting the importance of controlling for age in observational studies. In addition, these studies were based on relatively short-term effects of chemotherapy. It is possible that differential effects of these regimens on fatigue may be apparent further along in treatment, which is especially important, given that fatigue can continue beyond treatment [24]. In addition, the potential side effects of anthracycline-based chemotherapy remain of interest given their role in cardiotoxicity [18, 25].

The current analysis is based on data from the Understanding and Predicting Fatigue, Cardiovascular Decline and Events After Breast Cancer (UPBEAT) study of cardiovascular decline and fatigue following treatment for breast cancer. The current analyses examine whether women newly diagnosed with breast cancer who received anthracycline-based chemotherapy report greater fatigue than patients who receive other types of chemotherapy, or aromatase inhibitors (AIs) without chemotherapy, controlling for selection factors related to chemotherapy type.

## Methods

UPBEAT (WF-97415, funded via R01CA199167) is a multicenter prospective cohort of women diagnosed with stage I to III breast cancer conducted through the Wake Forest National Cancer Institute Community Oncology Research Program (NCORP) Research Base (UG1CA189824) and ECOG-ACRIN (UG1CA189828) under a National Cancer Institute CIRB-approved protocol (ClinicalTrials.gov Identifier NCT02791581). The study design and rationale along with detailed inclusion and exclusion criteria have been described previously [26]. All participants, including non-cancer controls and those with breast cancer, were 18 years of age or older. Eligible women with breast cancer included those with stage I to III disease (including inflammatory, newly diagnosed, or locally recurrent being treated with curative intent) scheduled to receive chemotherapy, and with an Eastern Cooperative Oncology Group (ECOG) performance status between 0 and 2 [27]. Exclusion criteria for UPBEAT included starting chemotherapy or AI treatment prior to baseline visit, having medical contraindications for undergoing cardiovascular magnetic resonance imaging, a left ventricular ejection fraction < 50% if previously assessed prior to treatment, current pregnancy or lactation, uncontrolled hypertension, or a pre-existing inflammatory disease (e.g., systemic lupus). A convenience sample of female family and friends, who were referred by patients; and had no history of cancer, breast surgery, or chemotherapy served as a control group.

For the purpose of these analyses, women diagnosed with breast cancer were categorized into the following three groups: 1) received anthracycline-based chemotherapy along with other chemotherapy agents (all received doxorubicin), 2) received only non-anthracycline-based chemotherapy (e.g., paclitaxel, cyclophosphamide, capecitabine, gemcitabine), and 3) received AIs only and no chemotherapy. AIs included anastrazole, letrozole, or exemestane. As described above, our fourth group was composed of women without a history of breast cancer.

### Procedure

Data were collected in-person at 3 time points: 1) baseline: post diagnosis, but before beginning chemotherapy, and 2) three and 3) twelve months later. All participants underwent blood draws at baseline (including hematocrit). Blood draws at 3 months and 12 months were conducted only in the breast cancer patients.

#### Outcome

Fatigue was measured by the Functional Assessment of Chronic Illness Therapy Fatigue scale (FACIT-fatigue) [28] at baseline and 3 and 12 months. Respondents rated the degree to which 13 items applied in the past 7 days using a 5-point scale. Scores ranged from 0–52, with higher scores indicating lower fatigue. The measure has excellent internal consistency (alpha = 0.93) and test–retest reliability (r = 0.90) [29]. From the continuous fatigue score we also created a dichotomous (yes/no) severe fatigue variable (severe fatigue was defined as a fatigue score below 30) [30]. Although initially developed as part of the Functional Assessment of Cancer Therapy (FACT) measurement system, the expanded FACIT System now encompasses other chronic diseases (www.facit.org). The FACIT-fatigue scale does not refer to cancer, has been used in other chronic disease populations, and is also appropriate for a general population [28]. Further, it has been shown to differentiate fatigue in cancer patients from fatigue experienced in the general population [31]. A review noted the FACIT-fatigue scale had robust psychometric data and easy administration [32].

#### Covariates

Covariates included in analyses were age, number of comorbidities, cancer stage; estrogen receptor (ER), progesterone receptor (PR), and HER2 receptor status; and timing of various treatments (AI therapy, radiation therapy, and surgery) with respect to study visit. We also examined neoadjuvant vs adjuvant status of chemotherapy in a sensitivity analysis including only those who received chemotherapy. Breast cancer stage was determined according to the 8th edition of the American Joint Committee on Cancer Staging Manual [33], and medical treatment was retrieved from patients’ medical records. Comorbidities were self-reported at baseline and included the following 17 conditions (women were asked to report yes/no/don’t know to ever receiving a diagnosis): diabetes, hypertension, hypercholesterolemia, coronary artery disease, anemia, migraine, osteoarthritis, rheumatoid arthritis, osteoporosis, thyroid disease, Parkinson’s disease, cognitive impairment, chronic obstructive pulmonary disease, heart failure, stroke, kidney disease, and other (i.e., non-breast) cancer. For purposes of presentation and inclusion in statistical models, we summarized the total number of comorbidities into three groups: 0, 1, and 2 or more as a measure of comorbidity burden [34]. To examine treatment variables, we classified women as yes/no with regard to beginning the treatment before baseline, between the baseline and 3-month visit, or between the 3 and 12 month visits.

#### Hematocrit

We examined values of hematocrit over time by group, and also examined the proportion of participants who would be classified as anemic (hematocrit < 37) due to the known association between anemia and fatigue, [https://www.merckmanuals.com/professional/hematology-and-oncology/approach-to-the-patient-with-anemia/evaluation-of-anemia].

### Statistical analyses

All analyses were carried out in SAS (version 9.4, Cary, NC). A two-sided alpha level of 0.05 was used to denote statistical significance. Descriptive statistics were computed for each of the four groups. We report baseline demographic and clinical characteristics, presenting frequencies and percentages for categorical variables and mean and standard deviation (or median and interquartile range if more appropriate) for continuous variables.

Self-reported comorbidities were not added to the baseline forms until after approximately 20% of the sample had already completed their baseline assessments. Given the central role of comorbidities in selection to chemotherapy regimen, the present analyses limit the sample to the approximately 80% of UPBEAT participants from whom self-reported comorbidity data were collected. There were no significant differences in age or cancer stage between those recruited before or after comorbidities were assessed.

To compare trends across time (baseline, 3 months, 12 months) in the four groups on the fatigue score and in proportion with severe fatigue, we estimated least squares means by group using mixed models with a random subject effect, fixed effects of time and group, and the interaction between time and group. For severe fatigue, these were means [proportions] of a 0/1 variable. Time was treated as a categorical (class) variable to avoid imposing assumptions about monotonic trends over time. For the modeling of the dichotomous severe fatigue outcome we used a repeated measures mixed model with a logit link function.

We ran two main adjusted models each for the fatigue score and for severe fatigue. The first model included all 4 groups and contained the above terms of group, time, group*time interaction, and age and comorbidity category as covariates. Age was included as a continuous variable, while comorbidity status was included as a categorical variable. The second adjusted model, run on the three cancer groups only, added the cancer and treatment variables: cancer stage; ER, PR, and HER2 status (positive or negative/unknown); and timing of radiation, surgery, and AI therapy (i.e., start before baseline, between baseline and 3 mo. visit, or between 3 and 12 month visits). Due to the small numbers of women on hormonal (e.g., tamoxifen) therapy, and the lack of variability in timing of chemotherapy start in each group, we did not control for timing of these treatments.

Finally, two sensitivity analyses were conducted. One was among the three cancer groups in which we included hematocrit values (or % anemic) in the adjusted model, and another was among the two chemotherapy groups alone, examining neoadjuvant vs adjuvant chemotherapy timing.

## Results

### Sample characteristics

Out of 403 women enrolled in UPBEAT, there were a total of n = 284 in our analytic sample. For the present analyses, we excluded women for the following reasons: lack of data on comorbidities (N = 85), and for cancer patients: no treatment data provided (N = 10), began chemotherapy before baseline visit (N = 11), began AIs before baseline (N = 4), and began both chemotherapy and AIs before baseline (N = 1). Finally, because of the known effect of radiation on fatigue, we also excluded 8 women who had radiation shortly before baseline. These exclusions resulted in a sample of n = 284.

Baseline and clinical characteristics of the four groups are shown in Table 1. The groups differed in age, with cancer patients who did not receive chemotherapy being the oldest, on average, and the non-cancer controls the youngest. The youngest cancer group were those who received anthracycline-based chemotherapy. The groups also differed on comorbidity status, with non-cancer controls having the fewest comorbidities and the cancer with no chemotherapy group (the oldest group) having the most. Among the three cancer groups, there was a significant difference in stage of cancer at diagnosis, with a shift towards higher stage for those receiving anthracycline-based chemotherapy and towards lower stage for those receiving an AI and no chemotherapy. At 3 and 12 months, 275 and 242 participants remained, respectively.

**Table 1:** Participant characteristics (*n* = 284)

Characteristic	Cancer with anthracycline chemotherapy (*n* = 98)	Cancer with other chemotherapy (*n* = 64)	Cancer with AI and no chemotherapy (*n* = 20)	Non-cancer controls (*n* = 102)	*p*
Age (years), mean (SD)	53.0 (10.8)	56.6 (11.1)	64.5 (6.7)	49.5 (12.5)	** < 0.0001**
Ethnicity					
Hispanic	1 (1.0)	1 (1.6)	0 (0)	2 (2.0)	0.87
Non-Hispanic	93 (94.9)	58 (90.6)	20 (100)	95 (93.1)	
Unknown	4 (4.1)	5 (7.8)	0 (0)	5 (4.9)	
Race					0.87
White	72 (73.5)	51 (79.7)	17 (85.0)	77 (75.5)	
Black or African-American	20 (20.4)	10 (15.6)	2 (10.0)	16 (15.7)	
Other	6 (6.1)	3 (4.7)	3 (11.1)	9 (9.1)	
Number of comorbidities					**0.047**
None	22 (22.5)	11 (17.2)	2 (10.0)	31 (30.4)	
1	31 (31.6)	17 (26.6)	3 (15.0)	32 (31.4)	
2 or more	45 (45.9)	36 (56.3)	15 (75.0)	39 (38.2)	
Five most prevalent self-reported comorbidities at baseline					
High cholesterol	25 (25.1)	25 (39.1)	13 (65.0)	30 (29.4)	**0.007**
Hypertension	23 (23.5)	24 (37.5)	10 (50.0)	21 (20.8)	**0.002**
Arthritis	23 (23.5)	16 (25.0)	7 (35.0)	13 (12.8)	**0.03**
Migraine	23 (23.5)	13 (20.3)	5 (25.0)	19 (18.6)	0.87
Anemia	19 (19.4)	15 (23.4)	6 (31.6)	16 (15.7)	0.43
Baseline % hematocrit, mean (SD)	38.8 (3.6)	39.7 (3.2)	40.0 (2.4)	40.2 (2.8)	**0.02**
Anemia defined as HCT<37 baseline	23 (23.5)	12 (19.1)	4 (16.0)	11 (11.8)	0.21
Cancer stage					** < 0.0001**
I	18 (18.4)	28 (43.8)	18 (90.0)	Na	
II	69 (70.4)	28 (43.8)	2 (10.0)	Na	
III	11 (11.2)	8 (12.5)	0 (0)	Na	
Receptor status					
ER +	48 (49.0)	47 (73.4)	20 (100)		** < 0.0001**
PR +	41 (41.8)	38 (59.4)	18 (90.0)		** < 0.0001**
HER2 +	6 (6.2)	43 (68.3)	1 (5.0)		** < 0.0001**

*p*-values < .05 are bolded

Among those who received anthracyclines (doxorubicin), 96.9% began chemotherapy after the baseline visit and before 3 months, while others began between the 3 and 12-month. visits; 78.6% began an additional chemotherapy agent before the 3-month visit (Table 2). Among those who received non-anthracycline chemotherapy only, 92.2% started chemotherapy before the 3-month visit. A significantly higher proportion of the anthracycline group received neoadjuvant chemotherapy compared to the other chemotherapy group (61% vs 37.7%, respectively; p = 0.004).

**Table 2:** Treatment type and timing (*n*, %)

Treatment characteristic	Cancer with anthracycline chemotherapy (*n* = 98)	Cancer with other chemotherapy (*n* = 64)	Cancer AI and no chemotherapy (*n* = 20)
Anthracycline (doxorubicin)			
Started between BL and 3 m visit	95 (96.9)	N/A	N/A
Started between 3-12 m visits	2^a^ (2.0)	N/A	N/A
Other chemotherapy			
Started between BL and 3 m visit	77 (78.6)	59 (92.2)	N/A
Started between 3-12 m visits	19 (19.4)	5 (7.8)	N/A
Began chemotherapy at least 2 weeks prior to surgery (neoadjuvant chemotherapy)	58 (61)	23 (37.7)	N/A
AI therapy			
Started between BL and 3 m visit	1 (1.0)	3 (4.7)	14 (70.0)
Started between 3- 12 m visits	24 (24.5)	23 (35.9)	3 (15.0)
Hormonal therapy (non AI)^b^			
Started between BL and 3 m visit	0	0	0
Started between 3—12 m visits	6 (6.1)	6 (9.4)	0
Radiation therapy			
Started between BL and 3 m visit	3 (3.1)	4 (6.3)	0
Started between 3—12 m visits	63 (64.3)	39 (60.9)	0
Surgery			
Before BL visit	37 (37.8)	36 (56.3)	18 (90.0)
Before 3 m visit	7 (7.1)	4 (6.3)	2 (10.0)
Before 12 m visit	46 (46.9)	16 (25.0)	0

*AI* aromatase inhibitor, *BL* Baseline

^a^Numbers do not total to 100% due to loss to follow-up of 1 person

^b^Includes tamoxifen, goserelin, triptorelin, and leuprolide. Of the 12 who began this class of therapy in our sample, 10 were on tamoxifen

Only 6 women in each chemotherapy group began hormonal therapy, with all starting between the 3- and 12- month visits and the majority of these 12 women (n = 10) took tamoxifen. A total of 109 women received radiation therapy and 93.6% began after the 3-month visit. No one in the no chemotherapy group received radiation therapy. There was no significant difference in the receipt of radiation therapy between the two chemotherapy groups.

### Fatigue scores

**Fig. 1:**
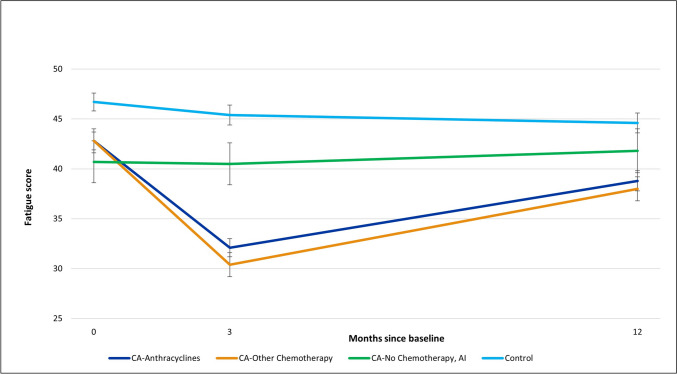
Estimated mean fatigue scores, with standard errors, by group and visit, adjusted for age, race, and comorbidities

In both unadjusted and adjusted models, there were highly significant overall group and time effects (*p* < 0.0001) in fatigue in the models with all four groups, with the three cancer groups having worse fatigue than the controls at each time point (Table 3, Fig. 1). Linear contrasts showed that at baseline, prior to any chemotherapy treatment, the three cancer groups did not differ from each other, but all had significantly lower scores (i.e., greater fatigue) than controls. They also had significantly worse fatigue than the controls at the other two time points. Only adjusted estimated means from the 4-group model containing age and comorbidity category are shown since they did not differ in any meaningful way from unadjusted estimated means and because both covariates of age and comorbidity status differed significantly across the four groups and were significantly associated with fatigue score.

**Table 3:** Estimated mean fatigue scores (and standard errors) by group and time, 4-group and 3-group adjusted models

Adjusted 4-group model^a^	Baseline	3 months	12 months
Cancer with anthracycline chemotherapy	42.8 (0.9)	32.1 (0.9)	38.8 (1.0)
Cancer with other chemotherapy	42.8 (1.2)	30.4 (1.2)	38.0 (1.2)
Cancer with AI, no chemotherapy	40.7 (2.1)	40.5 (2.1)	41.8 (2.2)
Non-Cancer controls	46.7 (0.9)	45.4 (1.0)	44.6 (1.0)
Adjusted 3-group model^b^			
Cancer with anthracycline chemotherapy	43.6 (1.2)	32.4 (1.2)	38.0 (1.4)
Cancer with other chemotherapy	44.0 (1.6)	30.6 (1.6)	37.5 (1.7)
Cancer with AI, no chemotherapy	40.9 (2.7)	41.5 (2.8)	41.3 (2.7)

lower fatigue scores indicate greater fatigue

^a^*p* values: group, time, and group*time interaction all *p*’s < 0.0001; age *p* = 0.018; comorbidity category *p* = 0.017

^b^*p* values: group *p* = 0.34; time *p* < 0.0001; group*time interaction *p* = 0.0002; age *p* = 0.07; comorbidity category *p* = 0.036; radiation timing *p* = 0.02; AI timing *p* = 0.16; surgery timing *p* = 0.14; ER, PR, and HER2 status all *p* > 0.49

There was also a significant (*p* < 0.0001) interaction between group and time, as evidenced by the sharp drop in mean fatigue score at 3 months for the two chemotherapy groups followed by a partial recovery at 12 months, in comparison to the relatively stable mean fatigue scores over time in the non-cancer controls. The cancer without chemotherapy group was also comparatively stable. Of most relevance to our main study question, linear contrasts of estimated mean fatigue scores showed that the two chemotherapy groups did not differ significantly from each other at any time point.

In the 3-group model with cancer patients only, adjusting for stage at diagnosis, tumor characteristics (ER, PR, and HER2 status), radiation, AI, and surgery timing, there was a main effect of time, and a significant group*time interaction, with patterns of means closely paralleling the findings described above. In this model with only three groups, however, there was no main effect of group, most likely due to the removal of the non-cancer control group.

Sensitivity analysis among the two groups with chemotherapy that additionally controlled for neoadjuvant (vs adjuvant) chemotherapy, along with timing of other treatments, again found that the two chemotherapy groups did not differ in estimated mean fatigue scores in any meaningful or statistically significant way at any time point. In the sensitivity analysis examining the effect of including time-varying values of hematocrit in the full 3-group model described above, differences in group means of fatigue over time were dampened, which is the expected consequence of adjusting for a mediating variable, but time and the group*time interaction remained significant (data not shown). This same result held when we included time varying proportion defined as anemic based on hematocrit values.

### Severe fatigue

Estimated proportions of those classified as severely fatigued showed similar results as the fatigue score means (Table 4), though in both the 4-group and 3-group models (adjusted for the same factors as listed above for fatigue score), the group*time interaction was not significant. The proportion of women classified as severely fatigued greatly increased in the two chemotherapy groups at 3 months, and then declined by 12 months. These groups did not differ from each other at any time point. The proportion of women classified as having severe fatigue was considerably lower, and relatively stable over time, in the no chemotherapy and non-cancer control groups.

**Table 4:** Estimated proportions with severe fatigue (and standard errors) by group and time, 4-group and 3-group adjusted models

	Baseline	3 months	12 months
Adjusted 4-group model^a^			
Cancer with anthracycline chemotherapy	0.12 (0.03)	0.40 (0.05)	0.19 (0.04)
Cancer with other chemotherapy	0.11 (0.04)	0.49 (0.06)	0.24 (0.05)
Cancer with AI, no chemotherapy	0.15 (0.09)	0.15 (0.09)	0.14 (0.08)
Non-cancer controls	0.03 (0.02)	0.03 (0.02)	0.06 (0.03)
Adjusted 3-group model^b^			
Cancer with anthracycline chemotherapy	0.13 (0.04)	0.44 (0.06)	0.20 (0.05)
Cancer with other chemotherapy	0.08 (0.03)	0.48 (0.08)	0.23 (0.06)
Cancer with AI, no chemotherapy	0.11 (0.07)	0.19 (0.11)	0.13 (0.09)

^a^*p* values: group *p* < 0.0001; time *p* = 0.03; group*time interaction *p* = 0.15; age *p* = 0.17; comorbidity status *p* = 0.15

^b^*p* values: group *p* = 0.56; time *p* = 0.10; group*time interaction *p* = 0.34; age *p* = 0.62; comorbidity category *p* = 0.47; radiation timing *p* = 0.02; AI timing *p* = 0.16; surgery timing *p* = 0.14; ER, PR, and HER2 status all *p*’s > 0.49

## Discussion

The current study demonstrated that breast cancer patients receiving anthracycline-based chemotherapy do not report greater fatigue during or after chemotherapy receipt than patients receiving non-anthracycline-based chemotherapy. Both groups of patients showed a significant increase in fatigue at 3 months during chemotherapy, which subsided at 12 months, but did not reach baseline levels. Both chemotherapy groups had greater fatigue at 3 and 12 months than BCS who did not receive chemotherapy, but did receive aromatase inhibitors.

Our results for the mean fatigue scores are very similar to other studies that have used the FACIT to measure fatigue among breast cancer patients receiving chemotherapy [9, 31, 35–37]. Cella and colleagues suggest that a difference of 3 points on the FACIT-fatigue measure is a clinically meaningful difference [38]. Using this criterion, there is no meaningful difference between the two chemotherapy groups at any time point, but there is a meaningful difference between BCS who received chemotherapy and those who did not at both 3 and 12 months, although the difference is lessened at 12 months. We observed a stable level of fatigue among the cancer controls who received AIs only, suggesting that these agents did not affect fatigue in our sample. This finding is consistent with clinical trials of endocrine therapy [39, 40]. Overall, these observations can inform expectations of some resilience to the symptoms of fatigue for women who receive chemotherapy. Further, the lack of difference in fatigue severity between women receiving anthracycline versus non-anthracycline is an important consideration in treatment decision-making.

Despite improvements in fatigue scores seen at 12 months, early interventions to mitigate fatigue severity could benefit women starting breast cancer chemotherapy. ASCO has previously recommended that that all patients with cancer be evaluated for fatigue after completion of primary treatment [2]. Our findings support the need for regular screening, assessment, education, and appropriate treatment for fatigue earlier in their treatment course. ASCO has suggested several treatment options that may help reduce fatigue among some patients. These include physical activity, psychosocial interventions, and mind–body interventions [2].

Several limitations of this study should be noted. As an observational study, we cannot rule out selection bias to treatment. Despite controlling for age and number of comorbidities, two factors used by clinicians in recommending treatment for breast cancer, there are other factors that clinicians consider that we did not control for such as performance status, physical activity, non-cancer medication use, or menopause status. Duration of chemotherapy treatment course is an additional factor that could influence fatigue. Because patients were not randomly assigned to cancer treatment group, caution must be exercised in attributing observed differences (or lack of differences) in fatigue to treatment regimen alone. It is possible that women who received anthracycline were healthier than those receiving alternate chemotherapy which may have attenuated differences between groups. Collection of patient-reported symptoms in randomized therapeutic trials are optimal to compare symptom side effects by regimen. Because part of this study occurred during COVID, some women were delayed in attending their 3-month visit. However, 85% of women had their 3-month follow-up within 4 months of baseline and over 95% had their 12 month visit within 16 months of baseline. Although doxorubicin was the only anthracycline used in this study, this reflects national treatment patterns. Despite these limitations, our study has several notable strengths, including a sample of well-characterized women recruited prior to treatment for breast cancer, the ability to control for important selection factors to chemotherapy type, and a non-cancer control group.

The present study supports previous studies showing significant fatigue among breast cancer patients treated with chemotherapy around the time of treatment, but some improvement by one year following the start of chemotherapy. Our study, over a longer time period than previous studies, further suggests no difference in fatigue between non- and anthracycline-based chemotherapy as patients in both groups had a similar fatigue trajectory.

## Data Availability

Data could be made available upon request to NCORP@wakehealth.edu.
